# Intestinal *Collinsella* may mitigate infection and exacerbation of COVID-19 by producing ursodeoxycholate

**DOI:** 10.1371/journal.pone.0260451

**Published:** 2021-11-23

**Authors:** Masaaki Hirayama, Hiroshi Nishiwaki, Tomonari Hamaguchi, Mikako Ito, Jun Ueyama, Tetsuya Maeda, Kenichi Kashihara, Yoshio Tsuboi, Kinji Ohno

**Affiliations:** 1 Department of Pathophysiological Laboratory Sciences, Nagoya University Graduate School of Medicine, Nagoya, Japan; 2 Division of Neurogenetics, Center for Neurological Diseases and Cancer, Nagoya University Graduate School of Medicine, Nagoya, Japan; 3 Division of Neurology and Gerontology, Department of Internal Medicine, School of Medicine, Iwate Medical University, Iwate, Japan; 4 Department of Neurology, Okayama Kyokuto Hospital, Okayama, Japan; 5 Department of Neurology, Fukuoka University, Fukuoka, Japan; INRAE, FRANCE

## Abstract

The mortality rates of COVID-19 vary widely across countries, but the underlying mechanisms remain unelucidated. We aimed at the elucidation of relationship between gut microbiota and the mortality rates of COVID-19 across countries. Raw sequencing data of 16S rRNA V3-V5 regions of gut microbiota in 953 healthy subjects in ten countries were obtained from the public database. We made a generalized linear model (GLM) to predict the COVID-19 mortality rates using gut microbiota. GLM revealed that low genus *Collinsella* predicted high COVID-19 mortality rates with a markedly low *p*-value. Unsupervised clustering of gut microbiota in 953 subjects yielded five enterotypes. The mortality rates were increased from enterotypes 1 to 5, whereas the abundances of *Collinsella* were decreased from enterotypes 1 to 5 except for enterotype 2. *Collinsella* produces ursodeoxycholate. Ursodeoxycholate was previously reported to inhibit binding of SARS-CoV-2 to angiotensin-converting enzyme 2; suppress pro-inflammatory cytokines like TNF-α, IL-1β, IL-2, IL-4, and IL-6; have antioxidant and anti-apoptotic effects; and increase alveolar fluid clearance in acute respiratory distress syndrome. Ursodeoxycholate produced by *Collinsella* may prevent COVID-19 infection and ameliorate acute respiratory distress syndrome in COVID-19 by suppressing cytokine storm syndrome.

## Introduction

COVID-19 is caused by severe acute respiratory syndrome coronavirus 2 (SARS-CoV-2). The infection has rapidly spread worldwide and has a great impact on medical care and the economy. SARS-CoV-2 causes widely variable phenotypes from lack of any symptoms, mild phenotype, rapidly progressive phenotype, to respiratory failure. The mortality rate increases exponentially with age with about one in ten patients over 80 years of age [1]. Risk factors associated with high mortality rates include obesity, diabetes, tobacco smoking, a past history of respiratory infection, and aging [2]. These risk factors should be similar among countries. However, there are large differences in mortality rates between countries. Mortality rates are higher in the United States, Europe, and South America than in Asia. In Europe, Spain and Italy have high mortality rates, whereas Germany and Northern Europe have low mortality rates (https://ourworldindata.org/). Similarly, in Asia, Taiwan and China have lower mortality rates than Japan and Korea. The differences could be accounted for by the differences in genome, previous exposure to less virulent corona virus, policies against COVID-19 pandemic, and/or gut microbiota.

A link between gut microbiota and COVID-19 has been postulated based on four observations [3]. First, chronic obstructive pulmonary disease (COPD) and inflammatory bowel diseases (IBDs) share similarities in epidemiology, clinical features, and inflammatory pathologies, which can be accounted for by gut dysbiosis, although other explanations are still possible [4]. Second, gut microbiota regulates the innate and adaptive immune system [5]. Third, germ-free mice, lacking of their gut bacteria, are not able to clear pathogen in the lungs [6]. Fourth, the removal of neomycin-sensitive gut bacteria in mice increases susceptibility to influenza virus infection [7]. Temporal profiles of gut microbiota in COVID-19 infection have been reported but without consistent bacterial features [8–11].

In the course of our analysis of gut microbiota in Parkinson’s disease [12] and idiopathic rapid-eye-movement sleep behavior disorder [13] in the world, we noticed that the mortality rates of COVID-19 may be associated with gut microbiota. We analyzed the relationship between the composition of intestinal bacteria in 953 healthy subjects in ten countries and the mortality rates of COVID-19 in these countries, and found that genus *Collinsella* was negatively correlated with the mortality rates of COVID-19.

## Results

### Generalized linear model (GLM) analysis

**Fig 1:**
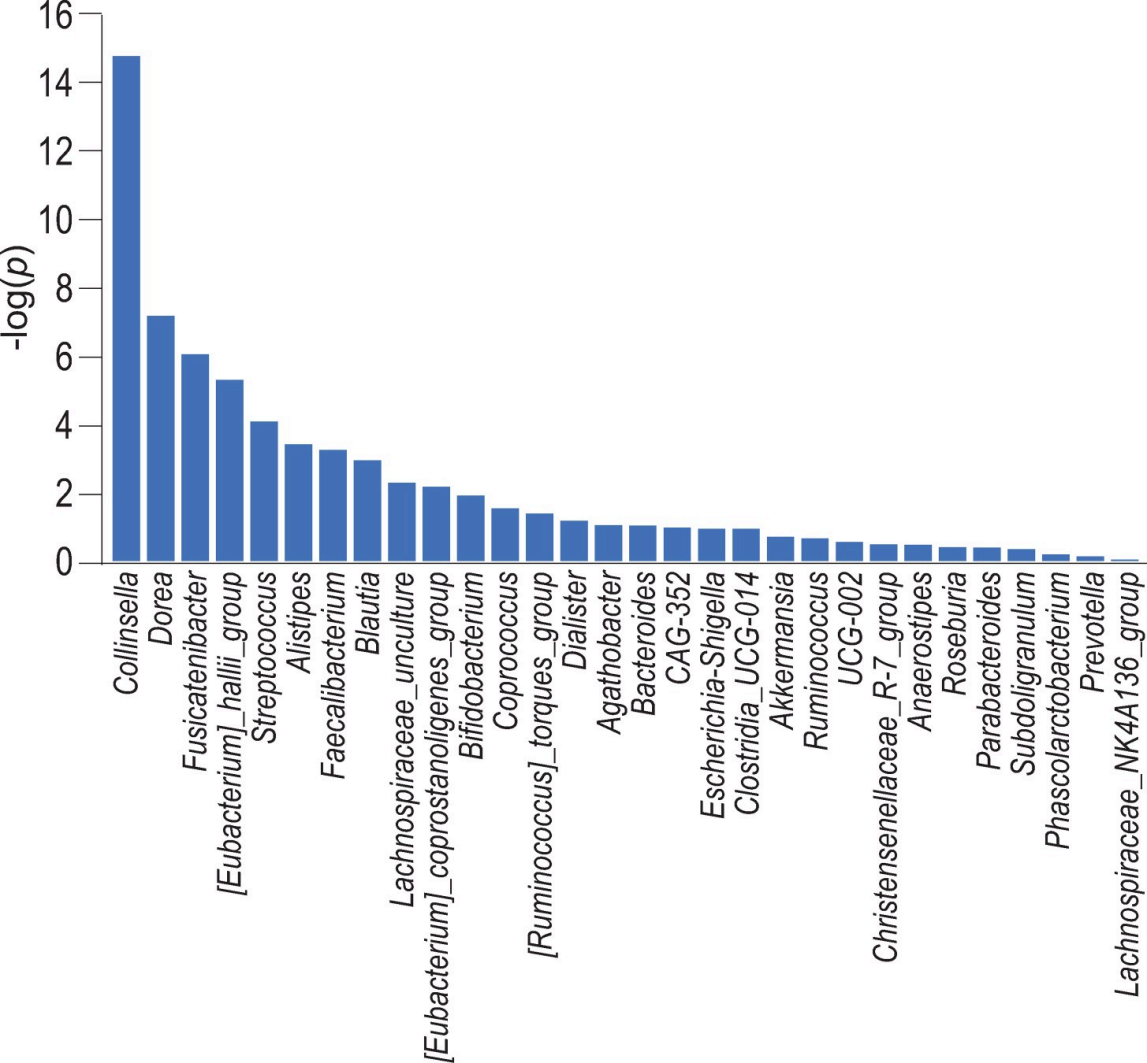
Plot of *p*-values of 30 genera in a generalized linear model (GLM) to predict the COVID-19 mortality rates.

To examine the effects of gut microbiota on the COVID-19 mortality rates across countries, we obtained 16S rRNA V3-V5 sequencing data of 953 healthy subjects in ten countries in the Organization for Economic Co-operation and Development (OECD) (Table 1), where high medical and hygienic standards were similarly expected with less biased geopolitical factors. We first analyzed relative abundance of each intestinal bacterium at the genus level in each subject. We then predicted the COVID-19 mortality rates in ten countries with the 30 most abundant genera using GLM. In GLM analysis, we compared gaussian, gamma, and inverse gaussian distributions, and found that gamma distribution gave rise to the lowest Akaike’s Information Criterion (AIC) (gaussian, 14599; gamma, 14573; and inverse gaussian, 15318). The results of GLM analysis using gamma distribution are plotted in Fig 1 and are indicated in S1 Table. Genus *Collinsella* had a marked negative predictive value for the COVID-19 mortality rates with the lowest *p*-value. Genera *Dorea* and *Fusicatenibacter*, which are short chain fatty acid (SCFA)-producing bacteria, also had high predictive values for the COVID-19 mortality rates with the second and third lowest *p*-values.

**Table 1:** Ten 16S rRNA-seq datasets from ten countries and the mortality rates of COVID-19.

Dataset	First author	City, Country	Accession number	Mortality rate per million^a^	The number of samples	Sequencing	Primers
1	Jung [14]	Daegu, South Korea	PRJNA644464	29.0	104	16S rRNA V4–V5	515F/907R
2	Nishiwaki [12]	Nagoya, Japan	DRA009229	52.2	137	16S rRNA V3-4	341F/805R
3	Aho [15]	Helsinki, Finland	PRJEB27564	126.9	64	16S rRNA V3-V4	341F1–4/785R1–4
4	Turpin [16]	Toronto, Canada	PRJEB14839	554.1	137^b^	16S rRNA V4	515F/806R
5	Heintz-Buschart [17]	Kassel, Germany	PRJNA381395	752.0	38	16S rRNA V4	515F/805R
6	Chávez-Carbajal [18]	Mexico City, Mexico	PRJNA417691	1306.4	25	16S rRNA V3	341F/518R
7	Hill-Burns [19]	Birmingham, AL, USA	ERP016332	1429.6	133	16S rRNA V4	515F/806R
8	Pietrucci [20]	Rome, Italy	PRJNA510730	1521.7	72	16S rRNA V3-V4	not specified
9	Jackson [21]	London, United Kingdom	PRJEB13747	1680.3	137^b^	16S rRNA V4	not specified
10	Vandeputte [22]	Leuven, Belgium	PRJEB21504	1852.7	106	16S rRNA V4	515F/806R

^a^The accumulated number of deaths per million people at https://ourworldindata.org/ on February 9, 2021, when vaccines were not widely used in these countries.

^b^For unbiased analysis, the numbers of samples were randomly reduced to 137 from 1561 in Canada and 2700 in United Kingdom.

### Linked Inference of Genomic Experimental Relationships (LIGER) analysis

**Fig 2:**
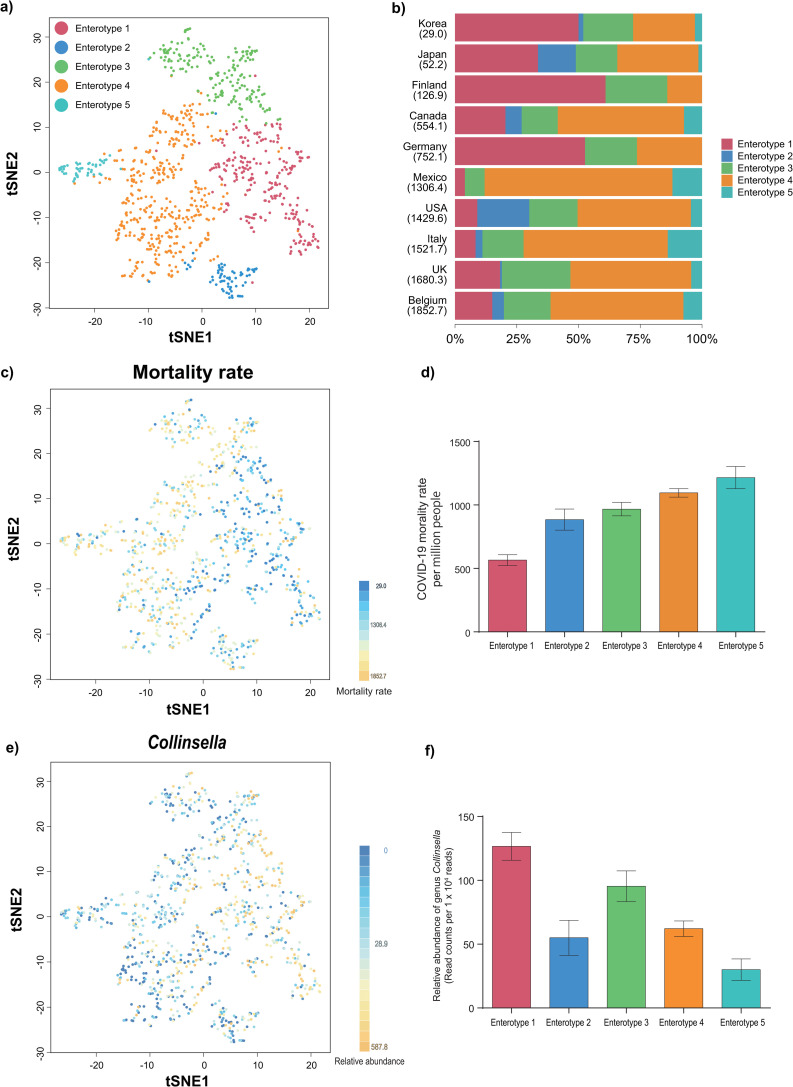
Five enterotypes in ten countries and their relevance to the COVID-19 mortality rates and genus *Collinsella*. **(a)** Unsupervised clustering of gut microbiota in 953 healthy subjects in ten countries by LIGER generated five enterotypes. Each subject is plotted with t-SNE and is color-coded by its enterotype. **(b)** Fractions of enterotypes 1 to 5 in ten countries. Ten countries are sorted in ascending order of the COVID-19 mortality rates per million, which are indicated in parentheses. **(c)** The t-SNE plot is color-coded by the COVID-19 mortality rates in ten countries. **(d)** Mean and standard error of the COVID-19 mortality rates in enterotypes 1 to 5. *P* = 2.2E-16 by Jonckheere-Terpstra trend test. **(e)** The t-SNE plot is color-coded by the relative abundance of genus *Collinsella*. **(f)** Mean and standard error of the relative abundance of genus *Collinsella* in enterotypes 1 to 5. *P* = 3.7E-12 by Jonckheere-Terpstra trend test. Color code in **(a)**, **(b)**, **(d)**, and **(f)** are matched.

Non-negative matrix factorization of gut microbiota in 953 healthy subjects in ten countries by a single cell RNA-seq analysis tool, LIGER [23], yielded five enterotypes (Fig 2A). The mean relative abundances of 30 most prevalent genera for each enterotype are collated in S2 Table. Ten countries were sorted in order of increasing COVID-19 mortality rates, and fractions of the five enterotypes were plotted in Fig 2B. The rates of enterotype 1 were high in countries where the mortality rates were low, whereas the rates of enterotypes 4 and 5 were high in countries where the mortality rates were high. Indeed, color-coding of the COVID-19 mortality rates on the LIGER plot showed that the mortality rates were increased from the right side to the left side (Fig 2C). The average mortality rates were increased form enterotypes 1 to 5 (Fig 2D). Color-coding of the relative abundance of genus *Collinsella* on the LIGER plot showed that *Collinsella* was decreased from the right side to the left side (Fig 2E). The average relative abundances of *Collinsella* were decreased form enterotypes 1 to 5 except for enterotype 2 (Fig 2F). Thus, in five enterotypes in ten countries, high *Collinsella* was predictive of low mortality rates of COVID-19. Plots of the average relative abundances of genera *Dorea* and *Fusicatenibacter* that had the second and third lowest *p*-values in GLM analysis (S1 Table) for each enterotype showed that genus *Dorea* was decreased from enterotypes 1 to 5 except for enterotype 2 (S1a Fig) and genus *Fusicatenibacter* was the highest in enterotype 1 (S1b Fig). Thus, genera *Dorea* and *Fusicatenibacter* were also predictive of mortality rates of COVID-19, although the *p*-values in GLM analysis were 4.20 x 10^7^ and 5.54 x 10^8^ times higher than that of genus *Collinsella*.

## Discussion

We made a machine-learning GLM to predict the COVID-19 mortality rates with gut microbiota in 953 healthy subjects in ten countries. Some of the 953 subjects might have been infected by SARS-CoV-2 and might have died, but anonymity of these subjects prevented us from tracing COVID-19 in these subjects. Even if we could trace COVID-19, the number of subjects was too low to analyze gut microbiota in fatal cases. However, as specific bacteria or enterotypes are enriched in specific countries [24, 25], we hypothesized that gut microbiota in healthy subjects in ten countries might account for the difference in widely variable COVID-19 mortality rates across countries. We found that genus *Collinsella* was negatively correlated with the mortality rate with a markedly low *p*-value of 1.58 x 10^−15^ (S1 Table and Fig 1). We next performed unsupervised clustering of gut microbiota in 953 healthy subjects using LIGER, and observed the presence of five enterotypes (Fig 2A). The mortality rates were increased (Fig 2B and 2D) and the relative abundances of genus *Collinsella* were decreased (Fig 2F) from enterotypes 1 to 5. SCFA-producing genera *Dorea* and *Fusicatenibacter* had the second and third lowest *p*-values in GLM analysis and highly correlated with the COVID-19 mortality rates (S1 Table and Fig 1). In accordance with our observations, analyses of gut microbiota in COVID-19 patients in Hong Kong [9] and Portugal [26] showed that genus *Collinsella* and SCFA-producing bacteria were decreased in severe COVID-19 patients compared to mild COVID-19 patients.

**Fig 3:**
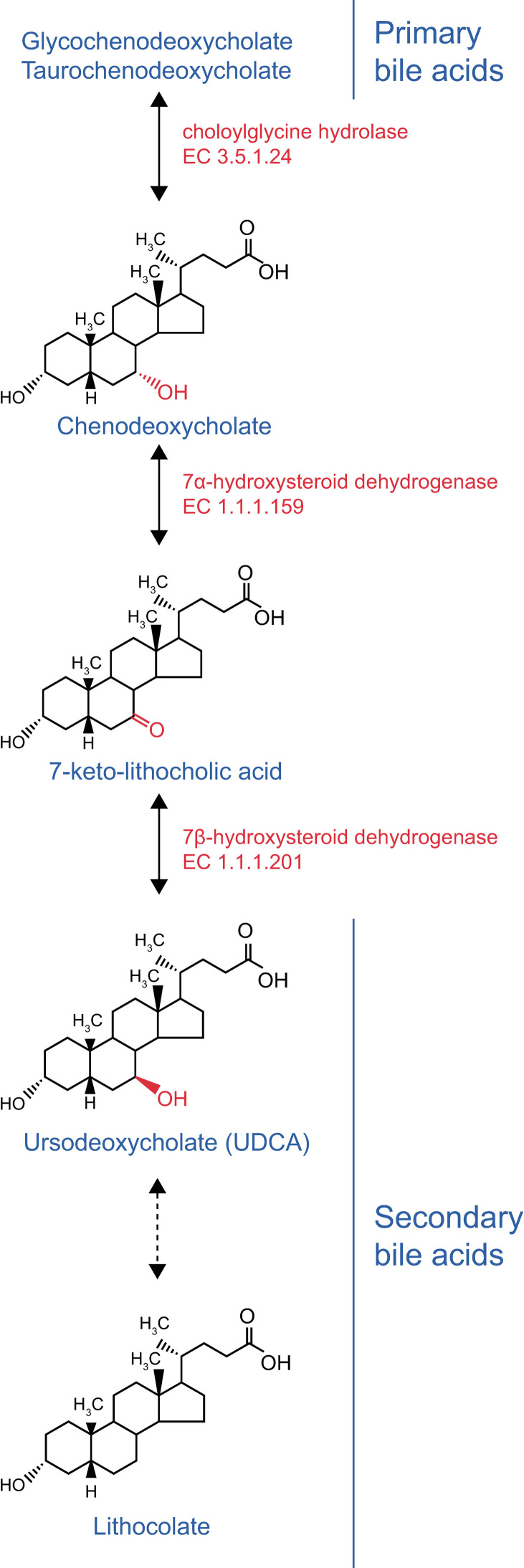
Excerpt of KEGG pathway “Secondary Bile Acid Biosynthesis”.

About 5% primary bile acids escape absorption in the small intestine and are biotransformed to secondary bile acids including ursodeoxycholic acid (UDCA) by intestinal bacteria [27]. Genus *Collinsella* carries a gene encoding an NADPH-dependent 7β-hydroxysteroid dehydrogenase (7β-HSDH) (Fig 3) [28], and is an essential intestinal bacterium to produce UDCA and other secondary bile acids. Three lines of evidence suggest that UDCA prevents SARS-CoV-2 infection and/or ameliorates COVID-19. First, docking simulation indicates that UDCA blocks binding of the spike region of SARS-Cov-2 and ACE2, and UDCA indeed prevents their interaction in a dose-dependent manner [29, 30]. Second, UDCA suppresses pro-inflammatory cytokines like TNF-α, IL-1β, IL-2, IL-4, and IL-6 at the mRNA and protein levels [31, 32]. UDCA also has an antioxidant effect as a remarkable scavenger [33]. UDCA additionally has an anti-apoptotic effect [34]. UDCA is thus expected to suppress the cytokine storm syndrome causing respiratory failure in COVID-19 [35, 36]. Third, UDCA increases alveolar fluid clearance in a rat model of acute respiratory distress syndrome (ARDS) via ALX/cAMP/PI3K pathway [37]. UDCA has been approved by the US Food and Drug Administration (FDA) and other countries for primary biliary cholangitis and other cholestatic disorders, and has no major adverse effects [38]. It is thus expected that UDCA prevents binding of SARS-CoV-2 to ACE2, and ameliorates COVID-19 by suppressing pro-inflammatory cytokines and by mitigating ARDS. Further prospective and/or retrospective study of COVID-19 patients is required to confirm whether genus *Collinsella* is protective against COVID-19 infection and mitigates ARDS in COVID-19.

## Materials and methods

### Datasets

The prevalences of COVID-19 are highly variable from country to country. The accurate numbers of COVID-19 patients are difficult to be estimated, because young subjects infected with SARS-CoV-2 tend to be asymptomatic and the chance of detection of SARS-CoV-2 infection is dependent on the accessibility to PCR test [39–44]. Compared to the number of COVID-19 patients, the number of deaths due to COVID-19 should be less biased by geopolitical factors. To further make geopolitical factors unbiased, we selected ten OECD countries, where the raw 16S rRNA V3-V5 sequencing data in normal subjects were available on public [Japan [12], Korea [14], Finland [15], Canada [16], Germany [17], Mexico [18], USA [19], Italy [20], UK [21], and Belgium [22]] (Table 1). OECD countries are expected to have high medical and hygienic standards, and dependable health statistics. As our analysis was inevitably biased by the number of samples in each country, we randomly selected 137 samples out of 1,561 samples in Canada and 2,700 samples in the United Kingdom. The total number of samples was 953 in ten countries. The datasets were not filtered by age or sex, because we hoped to analyze as many subjects as possible and because average gut microbiota is similar from ages 20 to 70 in each country [45]. The accumulated numbers of deaths per million people were obtained from Our World in Data (https://ourworldindata.org/) on February 9, 2021 (Table 1), when vaccines were not widely used in these countries.

### Taxonomic analysis of gut microbiota

Taxonomic analysis was performed using QIIME2 as previously described [12, 13]. Briefly, the FASTQ files were quality-filtered, and Amplicon Sequence Variants (ASVs) were yielded by DADA2. No sample was discarded during this step. For taxonomic determination, a trained reference was made from the SILVA taxonomy database release 138 by q2-feature-classifier.

### GLM analysis

We first used GLM to predict the mortality rates using gut microbiota of healthy subjects in ten countries. To make GLM, we used 30 most abundant intestinal genera. The variance inflation factor (VIF) was calculated for each pair of genera using the R package HH version 3.1–40. We confirmed that the VIFs were all less than 2, indicating that there was no multicollinearity among these genera. We used the “glm” function in R. We applied the gaussian, gamma, and inverse gaussian distributions to GLM, and adopted the distribution with the lowest AIC.

### LIGER analysis

We developed three topic-based tools to analyze gut microbiota and enterotypes [46–48], but we used LIGER, which was developed for single-cell RNA-seq analysis [23], to identify enterotypes in ten countries. We previously applied LIGER for intestinal enterotype analysis in Parkinson’s disease and rapid eye movement sleep behavior disorder [13]. We then analyzed correlation between the enterotypes and the mortality rates.

## Supporting information

S1 Fig Mean and standard error of genera *Dorea* and *Fusicatenibacter* mortality rates in enterotypes 1 to 5. *P* = 2.7E-13 and 5.2E-10 by Jonckheere-Terpstra trend test, respectively.
(DOCX)

S1 Table Generalized linear model (GLM) to predict the COVID-19 mortality rates with 30 intestinal bacteria. ^**a**^Average relative abundance in 953 healthy subjects in ten countries.
(DOCX)

S2 Table The mean relative abundances of 30 most prevalent genera for each enterotype. (DOCX)

## References

[1] O’Driscoll M, Ribeiro Dos Santos G, Wang L, Cummings DAT, Azman AS, Paireau J, et al. Age-specific mortality and immunity patterns of SARS-CoV-2. Nature. 2021;590(7844):140–5. Epub 2020/11/03. doi: 10.1038/s41586-020-2918-0 . 33137809

[2] Jordan RE, Adab P, Cheng KK. Covid-19: risk factors for severe disease and death. BMJ. 2020;368:m1198. Epub 2020/03/29. doi: 10.1136/bmj.m1198 . 32217618

[3] Dhar D, Mohanty A. Gut microbiota and Covid-19- possible link and implications. Virus Res. 2020;285:198018. Epub 2020/05/21. doi: 10.1016/j.virusres.2020.198018 ; PubMed Central PMCID: PMC7217790. 32430279 PMC7217790

[4] Keely S, Talley NJ, Hansbro PM. Pulmonary-intestinal cross-talk in mucosal inflammatory disease. Mucosal Immunol. 2012;5(1):7–18. Epub 2011/11/18. doi: 10.1038/mi.2011.55 ; PubMed Central PMCID: PMC3243663. 22089028 PMC3243663

[5] Negi S, Das DK, Pahari S, Nadeem S, Agrewala JN. Potential Role of Gut Microbiota in Induction and Regulation of Innate Immune Memory. Front Immunol. 2019;10:2441. Epub 2019/11/22. doi: 10.3389/fimmu.2019.02441 ; PubMed Central PMCID: PMC6842962. 31749793 PMC6842962

[6] Fagundes CT, Amaral FA, Vieira AT, Soares AC, Pinho V, Nicoli JR, et al. Transient TLR activation restores inflammatory response and ability to control pulmonary bacterial infection in germfree mice. J Immunol. 2012;188(3):1411–20. Epub 2012/01/03. doi: 10.4049/jimmunol.1101682 . 22210917

[7] Ichinohe T, Pang IK, Kumamoto Y, Peaper DR, Ho JH, Murray TS, et al. Microbiota regulates immune defense against respiratory tract influenza A virus infection. Proc Natl Acad Sci U S A. 2011;108(13):5354–9. Epub 2011/03/16. doi: 10.1073/pnas.1019378108 ; PubMed Central PMCID: PMC3069176. 21402903 PMC3069176

[8] Zuo T, Zhang F, Lui GCY, Yeoh YK, Li AYL, Zhan H, et al. Alterations in Gut Microbiota of Patients With COVID-19 During Time of Hospitalization. Gastroenterology. 2020;159(3):944–55 e8. Epub 2020/05/23. doi: 10.1053/j.gastro.2020.05.048 ; PubMed Central PMCID: PMC7237927. 32442562 PMC7237927

[9] Yeoh YK, Zuo T, Lui GC, Zhang F, Liu Q, Li AY, et al. Gut microbiota composition reflects disease severity and dysfunctional immune responses in patients with COVID-19. Gut. 2021;70(4):698–706. Epub 2021/01/13. doi: 10.1136/gutjnl-2020-323020 ; PubMed Central PMCID: PMC7804842. 33431578 PMC7804842

[10] Zuo T, Zhan H, Zhang F, Liu Q, Tso EYK, Lui GCY, et al. Alterations in Fecal Fungal Microbiome of Patients With COVID-19 During Time of Hospitalization until Discharge. Gastroenterology. 2020;159(4):1302–10 e5. Epub 2020/07/01. doi: 10.1053/j.gastro.2020.06.048 ; PubMed Central PMCID: PMC7318920. 32598884 PMC7318920

[11] Gu S, Chen Y, Wu Z, Chen Y, Gao H, Lv L, et al. Alterations of the Gut Microbiota in Patients With Coronavirus Disease 2019 or H1N1 Influenza. Clin Infect Dis. 2020;71(10):2669–78. Epub 2020/06/05. doi: 10.1093/cid/ciaa709 ; PubMed Central PMCID: PMC7314193. 32497191 PMC7314193

[12] Nishiwaki H, Ito M, Ishida T, Hamaguchi T, Maeda T, Kashihara K, et al. Meta-Analysis of Gut Dysbiosis in Parkinson’s Disease. Mov Disord. 2020;35(9):1626–35. Epub 2020/06/20. doi: 10.1002/mds.28119 . 32557853

[13] Nishiwaki H, Hamaguchi T, Ito M, Ishida T, Maeda T, Kashihara K, et al. Short-Chain Fatty Acid-Producing Gut Microbiota Is Decreased in Parkinson’s Disease but Not in Rapid-Eye-Movement Sleep Behavior Disorder. mSystems. 2020;5(6):e00797–20. Epub 2020/12/10. doi: 10.1128/mSystems.00797-20 ; PubMed Central PMCID: PMC7771407. 33293403 PMC7771407

[14] Jung Y, Tagele SB, Son H, Ibal JC, Kerfahi D, Yun H, et al. Modulation of Gut Microbiota in Korean Navy Trainees following a Healthy Lifestyle Change. Microorganisms. 2020;8(9). Epub 2020/08/23. doi: 10.3390/microorganisms8091265 ; PubMed Central PMCID: PMC7569816. 32825401 PMC7569816

[15] Aho VTE, Pereira PAB, Voutilainen S, Paulin L, Pekkonen E, Auvinen P, et al. Gut microbiota in Parkinson’s disease: Temporal stability and relations to disease progression. EBioMedicine. 2019;44:691–707. doi: 10.1016/j.ebiom.2019.05.064 ; PubMed Central PMCID: PMC6606744. 31221587 PMC6606744

[16] Turpin W, Espin-Garcia O, Xu W, Silverberg MS, Kevans D, Smith MI, et al. Association of host genome with intestinal microbial composition in a large healthy cohort. Nat Genet. 2016;48(11):1413–7. Epub 2016/10/28. doi: 10.1038/ng.3693 . 27694960

[17] Heintz-Buschart A, Pandey U, Wicke T, Sixel-Doring F, Janzen A, Sittig-Wiegand E, et al. The nasal and gut microbiome in Parkinson’s disease and idiopathic rapid eye movement sleep behavior disorder. Mov Disord. 2018;33(1):88–98. Epub 2017/08/27. doi: 10.1002/mds.27105 ; PubMed Central PMCID: PMC5811909. 28843021 PMC5811909

[18] Chavez-Carbajal A, Nirmalkar K, Perez-Lizaur A, Hernandez-Quiroz F, Ramirez-Del-Alto S, Garcia-Mena J, et al. Gut Microbiota and Predicted Metabolic Pathways in a Sample of Mexican Women Affected by Obesity and Obesity Plus Metabolic Syndrome. Int J Mol Sci. 2019;20(2). Epub 2019/01/24. doi: 10.3390/ijms20020438 ; PubMed Central PMCID: PMC6358992. 30669548 PMC6358992

[19] Hill-Burns EM, Debelius JW, Morton JT, Wissemann WT, Lewis MR, Wallen ZD, et al. Parkinson’s disease and Parkinson’s disease medications have distinct signatures of the gut microbiome. Mov Disord. 2017;32(5):739–49. Epub 2017/02/15. doi: 10.1002/mds.26942 ; PubMed Central PMCID: PMC5469442. 28195358 PMC5469442

[20] Pietrucci D, Cerroni R, Unida V, Farcomeni A, Pierantozzi M, Mercuri NB, et al. Dysbiosis of gut microbiota in a selected population of Parkinson’s patients. Parkinsonism Relat Disord. 2019;65:124–30. Epub 2019/06/09. doi: 10.1016/j.parkreldis.2019.06.003 . 31174953

[21] Jackson MA, Verdi S, Maxan ME, Shin CM, Zierer J, Bowyer RCE, et al. Gut microbiota associations with common diseases and prescription medications in a population-based cohort. Nat Commun. 2018;9(1):2655. Epub 2018/07/10. doi: 10.1038/s41467-018-05184-7 ; PubMed Central PMCID: PMC6037668. 29985401 PMC6037668

[22] Vandeputte D, Kathagen G, D’Hoe K, Vieira-Silva S, Valles-Colomer M, Sabino J, et al. Quantitative microbiome profiling links gut community variation to microbial load. Nature. 2017;551(7681):507–11. doi: 10.1038/nature24460 . 29143816

[23] Welch JD, Kozareva V, Ferreira A, Vanderburg C, Martin C, Macosko EZ. Single-Cell Multi-omic Integration Compares and Contrasts Features of Brain Cell Identity. Cell. 2019;177(7):1873–87 e17. doi: 10.1016/j.cell.2019.05.006 ; PubMed Central PMCID: PMC6716797. 31178122 PMC6716797

[24] Arumugam M, Raes J, Pelletier E, Le Paslier D, Yamada T, Mende DR, et al. Enterotypes of the human gut microbiome. Nature. 2011;473(7346):174–80. doi: 10.1038/nature09944 ; PubMed Central PMCID: PMC3728647. 21508958 PMC3728647

[25] Nishijima S, Suda W, Oshima K, Kim SW, Hirose Y, Morita H, et al. The gut microbiome of healthy Japanese and its microbial and functional uniqueness. DNA Res. 2016;23(2):125–33. Epub 2016/03/10. doi: 10.1093/dnares/dsw002 ; PubMed Central PMCID: PMC4833420. 26951067 PMC4833420

[26] Moreira-Rosario A, Marques C, Pinheiro H, Araujo JR, Ribeiro P, Rocha R, et al. Gut Microbiota Diversity and C-Reactive Protein Are Predictors of Disease Severity in COVID-19 Patients. Front Microbiol. 2021;12:705020. Epub 2021/08/06. doi: 10.3389/fmicb.2021.705020 ; PubMed Central PMCID: PMC8326578. 34349747 PMC8326578

[27] Wahlstrom A, Sayin SI, Marschall HU, Backhed F. Intestinal Crosstalk between Bile Acids and Microbiota and Its Impact on Host Metabolism. Cell Metab. 2016;24(1):41–50. Epub 2016/06/21. doi: 10.1016/j.cmet.2016.05.005 . 27320064

[28] Liu L, Aigner A, Schmid RD. Identification, cloning, heterologous expression, and characterization of a NADPH-dependent 7beta-hydroxysteroid dehydrogenase from Collinsella aerofaciens. Appl Microbiol Biotechnol. 2011;90(1):127–35. Epub 2010/12/25. doi: 10.1007/s00253-010-3052-y . 21181147

[29] Poochi SP, Easwaran M, Balasubramanian B, Anbuselvam M, Meyyazhagan A, Park S, et al. Employing bioactive compounds derived from Ipomoea obscura (L.) to evaluate potential inhibitor for SARS-CoV-2 main protease and ACE2 protein. Food Front. 2020. Epub 2020/08/25. doi: 10.1002/fft2.29 ; PubMed Central PMCID: PMC7361879. 32838301 PMC7361879

[30] Carino A, Moraca F, Fiorillo B, Marchiano S, Sepe V, Biagioli M, et al. Hijacking SARS-CoV-2/ACE2 Receptor Interaction by Natural and Semi-synthetic Steroidal Agents Acting on Functional Pockets on the Receptor Binding Domain. Front Chem. 2020;8:572885. Epub 2020/11/17. doi: 10.3389/fchem.2020.572885 ; PubMed Central PMCID: PMC7645072. 33195060 PMC7645072

[31] Ko WK, Lee SH, Kim SJ, Jo MJ, Kumar H, Han IB, et al. Anti-inflammatory effects of ursodeoxycholic acid by lipopolysaccharide-stimulated inflammatory responses in RAW 264.7 macrophages. PLoS One. 2017;12(6):e0180673. Epub 2017/07/01. doi: 10.1371/journal.pone.0180673 ; PubMed Central PMCID: PMC5493427. 28665991 PMC5493427

[32] Ko WK, Kim SJ, Jo MJ, Choi H, Lee D, Kwon IK, et al. Ursodeoxycholic Acid Inhibits Inflammatory Responses and Promotes Functional Recovery After Spinal Cord Injury in Rats. Mol Neurobiol. 2019;56(1):267–77. Epub 2018/04/25. doi: 10.1007/s12035-018-0994-z . 29691718

[33] Lapenna D, Ciofani G, Festi D, Neri M, Pierdomenico SD, Giamberardino MA, et al. Antioxidant properties of ursodeoxycholic acid. Biochem Pharmacol. 2002;64(11):1661–7. Epub 2002/11/14. doi: 10.1016/s0006-2952(02)01391-6 . 12429355

[34] Kim YJ, Jeong SH, Kim EK, Kim EJ, Cho JH. Ursodeoxycholic acid suppresses epithelial-mesenchymal transition and cancer stem cell formation by reducing the levels of peroxiredoxin II and reactive oxygen species in pancreatic cancer cells. Oncol Rep. 2017;38(6):3632–8. Epub 2017/11/14. doi: 10.3892/or.2017.6045 . 29130098

[35] Abdulrab S, Al-Maweri S, Halboub E. Ursodeoxycholic acid as a candidate therapeutic to alleviate and/or prevent COVID-19-associated cytokine storm. Med Hypotheses. 2020;143:109897. Epub 2020/06/09. doi: 10.1016/j.mehy.2020.109897 ; PubMed Central PMCID: PMC7261102 competing financial interests or personal relationships that could have appeared to influence the work reported in this paper. 32505909 PMC7261102

[36] Subramanian S, Iles T, Ikramuddin S, Steer CJ. Merit of an Ursodeoxycholic Acid Clinical Trial in COVID-19 Patients. Vaccines (Basel). 2020;8(2). Epub 2020/06/25. doi: 10.3390/vaccines8020320 ; PubMed Central PMCID: PMC7350268. 32575350 PMC7350268

[37] Niu F, Xu X, Zhang R, Sun L, Gan N, Wang A. Ursodeoxycholic acid stimulates alveolar fluid clearance in LPS-induced pulmonary edema via ALX/cAMP/PI3K pathway. J Cell Physiol. 2019;234(11):20057–65. Epub 2019/04/12. doi: 10.1002/jcp.28602 . 30972764

[38] Bowlus CL, Kenney JT, Rice G, Navarro R. Primary Biliary Cholangitis: Medical and Specialty Pharmacy Management Update. J Manag Care Spec Pharm. 2016;22(10-a-s Suppl):S3–S15. Epub 2016/10/05. doi: 10.18553/jmcp.2016.22.10-a-s.s3 . 27700211 PMC10408407

[39] Mizumoto K, Kagaya K, Zarebski A, Chowell G. Estimating the asymptomatic proportion of coronavirus disease 2019 (COVID-19) cases on board the Diamond Princess cruise ship, Yokohama, Japan, 2020. Euro Surveill. 2020;25(10). Epub 2020/03/19. doi: 10.2807/1560-7917.ES.2020.25.10.2000180 ; PubMed Central PMCID: PMC7078829. 32183930 PMC7078829

[40] Kimball A, Hatfield KM, Arons M, James A, Taylor J, Spicer K, et al. Asymptomatic and Presymptomatic SARS-CoV-2 Infections in Residents of a Long-Term Care Skilled Nursing Facility—King County, Washington, March 2020. MMWR Morb Mortal Wkly Rep. 2020;69(13):377–81. Epub 2020/04/03. doi: 10.15585/mmwr.mm6913e1 ; PubMed Central PMCID: PMC7119514 Journal Editors form for disclosure of potential conflicts of interest. No potential conflicts of interest were disclosed. 32240128 PMC7119514

[41] Nishiura H, Kobayashi T, Miyama T, Suzuki A, Jung SM, Hayashi K, et al. Estimation of the asymptomatic ratio of novel coronavirus infections (COVID-19). Int J Infect Dis. 2020;94:154–5. Epub 2020/03/18. doi: 10.1016/j.ijid.2020.03.020 ; PubMed Central PMCID: PMC7270890. 32179137 PMC7270890

[42] Epidemiology Working Group for Ncip Epidemic Response CCfDC, Prevention. [The epidemiological characteristics of an outbreak of 2019 novel coronavirus diseases (COVID-19) in China]. Zhonghua Liu Xing Bing Xue Za Zhi. 2020;41(2):145–51. Epub 2020/02/18. doi: 10.3760/cma.j.issn.0254-6450.2020.02.003 . 32064853

[43] Lu X, Zhang L, Du H, Zhang J, Li YY, Qu J, et al. SARS-CoV-2 Infection in Children. N Engl J Med. 2020;382(17):1663–5. Epub 2020/03/19. doi: 10.1056/NEJMc2005073 ; PubMed Central PMCID: PMC7121177. 32187458 PMC7121177

[44] Ki M, Task Force for -nCo V. Epidemiologic characteristics of early cases with 2019 novel coronavirus (2019-nCoV) disease in Korea. Epidemiol Health. 2020;42:e2020007. Epub 2020/02/10. doi: 10.4178/epih.e2020007 ; PubMed Central PMCID: PMC7285424. 32035431 PMC7285424

[45] Odamaki T, Kato K, Sugahara H, Hashikura N, Takahashi S, Xiao JZ, et al. Age-related changes in gut microbiota composition from newborn to centenarian: a cross-sectional study. BMC Microbiol. 2016;16:90. Epub 2016/05/26. doi: 10.1186/s12866-016-0708-5 ; PubMed Central PMCID: PMC4879732. 27220822 PMC4879732

[46] Abe K, Hirayama M, Ohno K, Shimamura T. A latent allocation model for the analysis of microbial composition and disease. BMC Bioinformatics. 2018;19(Suppl 19):519. Epub 2019/01/02. doi: 10.1186/s12859-018-2530-6 ; PubMed Central PMCID: PMC6311924. 30598099 PMC6311924

[47] Abe K, Hirayama M, Ohno K, Shimamura T. ENIGMA: an enterotype-like unigram mixture model for microbial association analysis. BMC Genomics. 2019;20(Suppl 2):191. Epub 2019/04/11. doi: 10.1186/s12864-019-5476-9 ; PubMed Central PMCID: PMC6456936. 30967109 PMC6456936

[48] Abe K, Hirayama M, Ohno K, Shimamura T. Hierarchical non-negative matrix factorization using clinical information for microbial communities. BMC Genomics. 2021;22(1):104. Epub 2021/02/06. doi: 10.1186/s12864-021-07401-y ; PubMed Central PMCID: PMC7863378. 33541264 PMC7863378

