# Supplementary material for: Intestinal Collinsella may mitigate infection and exacerbation of COVID-19 by producing ursodeoxycholate
Source: PLoS One. 2021 Nov 23;16(11):e0260451. doi: 10.1371/journal.pone.0260451 (PMC8610263; doi:10.1371/journal.pone.0260451)
Supplement: S1 Fig — P = 2.7E-13 and 5.2E-10 by Jonckheere-Terpstra trend test, respectively. (DOCX) [file pone.0260451.s001.docx]

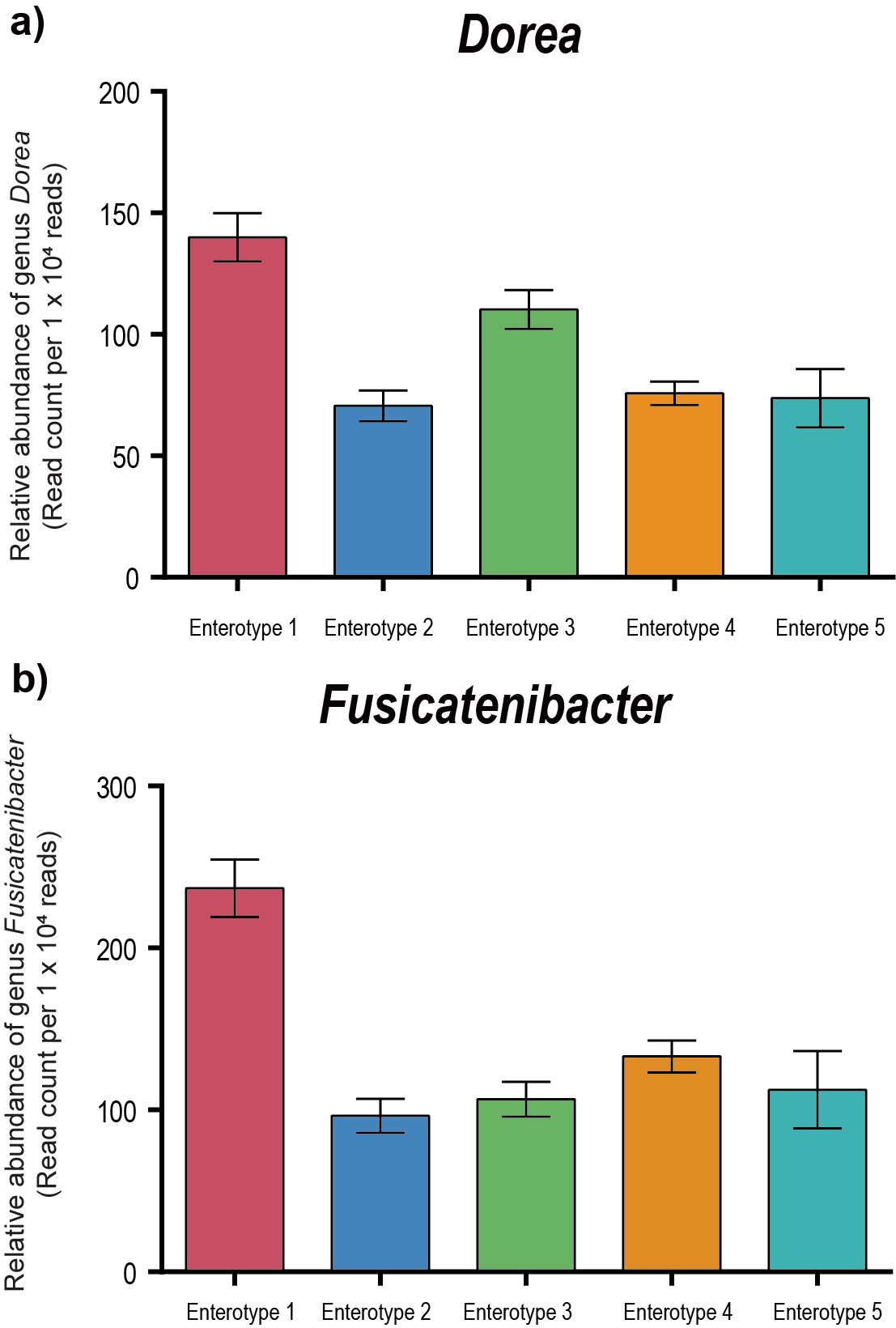


**Supplementary Figure S1.** Mean and standard error of genera *Dorea* and *Fusicatenibacter* mortality rates in enterotypes 1 to 5. *P* = 2.7E-13 and 5.2E-10 by Jonckheere-Terpstra trend test, respectively.
