# Supplementary material for: Intestinal Collinsella may mitigate infection and exacerbation of COVID-19 by producing ursodeoxycholate
Source: PLoS One. 2021 Nov 23;16(11):e0260451. doi: 10.1371/journal.pone.0260451 (PMC8610263; doi:10.1371/journal.pone.0260451)
Supplement: S1 Table — aAverage relative abundance in 953 healthy subjects in ten countries. (DOCX) [file pone.0260451.s002.docx]

**Supplementary Table 1. Generalized linear model (GLM) to predict the COVID-19 mortality rates with 30 intestinal bacteria**

| **Genera** | **Positive or negative effect** | ***P*-value** | **Relative abundance (%)^a^** |
| --- | --- | --- | --- |
| *Collinsella* | - | 1.58E-15 | 0.831 |
| *Dorea* | + | 6.63E-08 | 0.907 |
| *Fusicatenibacter* | - | 8.75E-07 | 1.38 |
| *[Eubacterium] hallii group* | + | 4.90E-06 | 0.858 |
| *Streptococcus* | - | 8.15E-05 | 1.06 |
| *Alistipes* | + | 3.81E-04 | 2.79 |
| *Faecalibacterium* | + | 5.59E-04 | 7.93 |
| *Blautia* | - | 1.13E-03 | 4.77 |
| *Lachnospiraceae unculture* | + | 5.11E-03 | 2.01 |
| *[Eubacterium]_coprostanoligenes group* | + | 6.80E-03 | 1.27 |
| *Bifidobacterium* | - | 0.0121 | 2.49 |
| *Coprococcus* | + | 0.0291 | 1.21 |
| *[Ruminococcus] torques group* | - | 0.0413 | 1.12 |
| *Dialister* | + | 0.0667 | 1.02 |
| *Agathobacter* | + | 0.0892 | 2.89 |
| *Bacteroides* | + | 0.0930 | 18.1 |
| *CAG-352* | + | 0.107 | 1.33 |
| *Escherichia-Shigella* | + | 0.114 | 2.47 |
| *Clostridia UCG-014* | + | 0.115 | 1.24 |
| *Akkermansia* | + | 0.199 | 1.17 |
| *Ruminococcus* | - | 0.217 | 1.37 |
| *UCG-002* | - | 0.276 | 1.97 |
| *Christensenellaceae R-7 group* | - | 0.326 | 1.71 |
| *Anaerostipes* | - | 0.333 | 1.06 |
| *Roseburia* | + | 0.399 | 1.62 |
| *Parabacteroides* | + | 0.409 | 1.43 |
| *Subdoligranulum* | - | 0.450 | 3.10 |
| *Phascolarctobacterium* | - | 0.644 | 0.808 |
| *Prevotella* | - | 0.736 | 4.78 |
| *Lachnospiraceae NK4A136 group* | + | 0.913 | 0.926 |

**^a^**Average relative abundance in 953 healthy subjects in ten countries
