# Supplementary material for: Intestinal Collinsella may mitigate infection and exacerbation of COVID-19 by producing ursodeoxycholate
Source: PLoS One. 2021 Nov 23;16(11):e0260451. doi: 10.1371/journal.pone.0260451 (PMC8610263; doi:10.1371/journal.pone.0260451)
Supplement: S2 Table — (DOCX) [file pone.0260451.s003.docx]

**Supplementary Table 2. The mean relative abundances of 30 most prevalent genera for each enterotype**

| Genus | Enterotype 1 | Enterotype 2 | Enterotype 3 | Enterotype 4 | Enterotype 5 |
| --- | --- | --- | --- | --- | --- |
| *Bacteroides* | 10.3% | 19.5% | 11.9% | 24.1% | 29.5% |
| *Faecalibacterium* | 9.2% | 5.5% | 6.0% | 8.5% | 7.5% |
| *Prevotella* | 8.4% | 4.9% | 2.7% | 3.9% | 1.3% |
| *Blautia* | 6.8% | 2.7% | 3.9% | 4.5% | 3.4% |
| *Subdoligranulum* | 4.2% | 1.7% | 3.3% | 2.6% | 2.8% |
| *Agathobacter* | 4.1% | 1.9% | 1.8% | 2.9% | 2.5% |
| *Alistipes* | 1.4% | 1.2% | 3.9% | 3.2% | 4.3% |
| *Bifidobacterium* | 3.4% | 3.2% | 2.3% | 2.1% | 0.8% |
| *Escherichia-Shigella* | 0.7% | 11.6% | 2.5% | 2.3% | 0.1% |
| *Lachnospiraceae unculture* | 1.6% | 1.7% | 1.3% | 2.5% | 3.1% |
| *UCG-002* | 1.9% | 0.9% | 4.0% | 1.4% | 1.4% |
| *Christensenellaceae_R-7_group* | 1.8% | 0.4% | 4.0% | 0.9% | 1.1% |
| *Roseburia* | 1.8% | 1.0% | 1.0% | 1.8% | 2.0% |
| *Parabacteroides* | 0.8% | 2.1% | 1.2% | 1.7% | 2.4% |
| *Fusicatenibacter* | 2.1% | 0.8% | 1.0% | 1.2% | 1.0% |
| *Ruminococcus* | 1.5% | 0.7% | 1.5% | 1.3% | 1.6% |
| *CAG-352* | 1.5% | 0.7% | 1.5% | 1.2% | 1.5% |
| *[Eubacterium]_coprostanoligenes_group* | 1.1% | 0.5% | 2.7% | 0.9% | 0.6% |
| *Clostridia_UCG-014* | 0.8% | 0.4% | 3.1% | 0.8% | 0.7% |
| *Coprococcus* | 1.7% | 0.6% | 1.5% | 0.9% | 1.0% |
| *Akkermansia* | 0.5% | 0.9% | 2.3% | 1.1% | 0.9% |
| *[Ruminococcus]_torques_group* | 1.4% | 0.6% | 1.1% | 1.1% | 0.7% |
| *Anaerostipes* | 1.7% | 0.6% | 0.7% | 1.0% | 0.5% |
| *Streptococcus* | 1.3% | 3.4% | 0.6% | 0.8% | 0.4% |
| *Dialister* | 1.0% | 1.0% | 0.8% | 1.2% | 0.3% |
| *NK4A136_group* | 0.9% | 0.6% | 0.9% | 1.0% | 1.4% |
| *Dorea* | 1.3% | 0.6% | 0.8% | 0.8% | 0.6% |
| *[Eubacterium]_hallii_group* | 1.2% | 0.4% | 0.8% | 0.8% | 0.5% |
| *Collinsella* | 1.3% | 0.6% | 1.0% | 0.6% | 0.3% |
| *Phascolarctobacterium* | 0.7% | 0.5% | 0.7% | 0.8% | 1.8% |
